# Outcomes in prenatally suspected focal placenta accreta spectrum

**DOI:** 10.1002/pmf2.70144

**Published:** 2025-11-06

**Authors:** Alesha White, Jessica E. Pruszynski, Quyen N. Do, Jennifer D. Smith, Catherine Y. Spong, Christina L. Herrera

**Affiliations:** 1 Department of Obstetrics & Gynecology, University of Texas Southwestern Medical Center, Dallas, Texas, USA; 2 Department of Obstetrics & Gynecology, Parkland Health, Dallas, Texas, USA; 3 Department of Radiology, University of Texas Southwestern Medical Center, Dallas, Texas, USA

**Keywords:** delivery timing, focal placenta accreta, placenta accreta spectrum

## Abstract

**Introduction:**

Morbidity of placenta accreta spectrum (PAS) increases with increasing severity of placental attachment. PAS with only a focal area of invasion or focal PAS based on magnetic resonance imaging (MRI) has been defined in a previous study as the greatest dimension of involvement of 25 mm or less. The objective of this study was to describe maternal and neonatal outcomes in patients with prenatally suspected focal PAS as defined by MRI imaging.

**Methods:**

This is a retrospective cohort study evaluating prenatally suspected focal PAS between 2020 and 2024. Focal PAS was defined based on MRI. Delivery and neonatal outcomes were described for the entire cohort and these outcomes were then compared between patients who delivered at <37 weeks (preterm) or at ≥37 weeks (term). A neonatal composite of cord pH < 7, cord base excess <−12, APGARs ≤3, continuous positive airway pressure/ventilator support, hypoxic ischemic encephalopathy, birthweight <3rd percentile, neonatal intensive care unit admission (NICU), and hypoglycemia was compared.

**Results:**

Of 40 cases of prenatally suspected focal PAS, 21 (52%) cases delivered preterm and 19 (48%) delivered at term. Six (15%) met International Federation of Gynecology and Obstetrics (FIGO) clinical criteria for PAS. Hysterectomy was performed in 3 (8%) cases. Pathology was negative in 33 (85%) patients, not sent in 1 patient, and positive for PAS in 6. The neonatal composite occurred in 11 (28%) neonates. Median APGARs at 1 min were 6 (6–8), no neonate had a pH < 7, and median birthweight was 2905 g (2721–3108 g). Ten (25%) infants were admitted to the NICU. Patients delivered preterm were more likely to have a placenta previa (76% vs. 42%, *p* = 0.0115) and were more likely to stay for more than 3 days in the hospital (24% vs. 0%, *p* = 0.049). Neonatal length of stay was longer (6 [4–14] vs. 4 [4–4] days, *p* = 0.003), and the rate of the neonatal composite outcome was higher in patients delivered preterm (43% vs. 11%, *p* = 0.034).

**Conclusion:**

Delivery and neonatal outcomes were favorable in cases of prenatally suspected focal PAS with low overall hysterectomy rates. Term delivery did not impact maternal outcomes, improved neonatal outcomes, and can be considered in an otherwise uncomplicated patient.

## 1 | INTRODUCTION

Placenta accreta spectrum (PAS) is associated with increased maternal morbidity and mortality and is the leading cause of peripartum hysterectomy [1–3]. Morbidity has been shown to correlate with increasing PAS severity, often secondary to associated increasing rate of hysterectomy [1, 2]. At our institution prior to 2022, there was no standardized timing for delivery of patients with prenatally suspected PAS with a focal area of invasion or focal PAS. We standardized delivery after 2022, with scheduled deliveries between 37 0/7 and 37 6/7 weeks in patients with concern for focal PAS based on subjective interpretation of ultrasound and magnetic resonance imaging (MRI) findings, given the internally observed reduced rate of hysterectomy in the focal PAS cohort. In 2024, our institution further established criteria for diagnosis of focal PAS using MRI to less than 25 mm, as the largest measurement of either the (1) placental or uterine bulge size, (2) length of involvement along the bladder–serosal interface, or (3) length of the location of dark T2 linear bands [4]. In a prior study, we found that a greatest dimension of invasion of 25 mm or less on MRI was associated with a 96% specificity for hysterectomy, an improvement from the use of our previous ultrasound-based risk assessment tool (the Placenta Accreta Index or PAI) alone [4, 5]. Currently, we use both MRI and clinical findings to dictate delivery timing in these patients. Clinical findings that are used to determine delivery timing include the presence of vaginal bleeding, preterm labor, and other obstetric indications that may warrant delivery earlier than 37 0/7. The purpose of this study is to describe the outcomes in cases of prenatally suspected focal PAS with emphasis on comparison of outcomes depending on time of delivery before 37 weeks (preterm) or at 37 weeks or greater (term).

## 2 | MATERIALS AND METHODS

This is a retrospective cohort study evaluating prenatally suspected focal PAS cases between 2020 and 2024. At our institution, all patients with a history of prior cesarean delivery (CD) and low-lying placenta or placenta previa are screened with ultrasound for PAS at the time of their anatomy scan (18–22 weeks) with application of the PAI [5]. Due to the potential for trophotrophism, a repeat sonogram is performed at 28 weeks. If there is continued concern for PAS at this scan via the application of the PAI, the patient is scheduled for an MRI scan at 30–32 weeks. On occasion, patients may also be brought to attention due to alternative risk factors for PAS (myomectomy, dilation, and curettage) and ultrasound findings concerning for PAS (previa and lacunae) and referred for MRI. Focal PAS was defined as less than 25 mm by the greatest dimension of involvement by MRI [4]. Only patients with a diagnosis of focal PAS based on this MRI definition were included in this study.

Patients with prenatally suspected focal PAS were admitted the day prior to their delivery for preoperative coordination. At the time of the surgery, a vertical hysterotomy was performed with consideration of the location of the placenta. It was left to the discretion of the maternal-fetal medicine providers to attempt removal of the placenta if possible. Following the formation of a multidisciplinary team beginning in late 2021 during scheduled cases, gynecology oncology surgeons were readily available if needed. Patients standardly recovered postoperatively in our step-down unit unless ICU care was required (ventilatory or pressure support).

Delivery and neonatal outcomes were evaluated for the entire group and then compared between term and preterm delivery to evaluate optimal delivery timing. Delivery outcomes assessed included rate of hysterectomy, placental bed oversewn, estimated blood loss (EBL), postpartum hemorrhage (PPH), blood products transfused, and length of hospital stay. Neonatal outcomes assessed included birthweight, rate of neonatal intensive care unit (NICU) admission, rate of ventilatory or continuous positive airway pressure (CPAP) support, and neonatal length of stay. The rate of a neonatal composite of cord pH < 7, cord base excess <−12, APGARs ≤3, CPAP/ventilator support, hypoxic ischemic encephalopathy, birthweight <3rd percentile, NICU admission, and hypoglycemia was calculated and compared between the two groups. Statistical analysis included the chi-square and Fisher’s exact tests for categorical variables, Student’s *t*-tests for normally distributed continuous variables, and Kruskal–Wallis test for non-normally distributed continuous variables. This study involves data retrospectively collected from human subjects. This study was approved by the institutional review board at our institution and the requirement for informed consent was waived.

## 3 | RESULTS

Of 40 prenatally suspected focal accreta cases, 24 (60%) patients had a placenta previa, 4 (10%) had a low-lying placenta, and 12 (30%) had a normal placental location at the time of delivery (Table 1). Of the 12 patients with a normal placental location at the time of delivery, 6 (50%) previously had a placental previa and 5 (42%) previously had a low-lying placenta in the index pregnancy. The one patient with a normal placental location at their initial scan and at the time of delivery was screened for PAS secondary to a history of two prior CDs and evidence of Grade 3 lacunae on the initial scan. All but one patient had a history of prior CD. Twenty-six (65%) deliveries were scheduled. As would be anticipated, more unscheduled cases occurred at <37 weeks (52% vs. 16%, *p* = 0.0219). Thirty (75%) patients had PPH and 14 (35%) received a transfusion (Table 2).

Six (15%) met International Federation of Gynecology and Obstetrics (FIGO) clinical criteria for PAS. Hysterectomy was performed in 3 (8%) cases and 6 (15%) cases required oversewing of the placental bed (Table 2). Gynecology oncology was planned to be readily available for delivery for 33 (83%) cases. For two of these cases gynecology oncology was present at the start of the procedure due to faculty preference. Two cases required assistance surgically from gynecology oncology, given the complexity of the case (bowel adhesions, maternal amniotic fluid embolism [AFE]), but not for PAS specifically. One (2.5%) patient was admitted to the intensive care unit after delivery secondary to AFE. Pathology was negative in 33 (85%) patients, not sent in 1 patient, and positive for PAS in 6 (3 via hysterectomy specimen, 3 via placental specimen based on basal plate myometrial fibers without intervening decidua) (Table 1). Of the three hysterectomy specimens, PAS was not clinically suspected in one case but rather AFE was the indication for hysterectomy. In the others, clinical FIGO Grade 1 and Grade 2 were diagnosed at the time of surgery. Pathology was positive for Grades 2, 3A, and 3E, respectively. Two of the six (33%) patients with a positive pathology for PAS had a normal placental location at the time of delivery. Of the three hysterectomy specimens with evidence of involvement, the greatest dimension of involvement on MRI was 15, 16, and 23 mm, respectively, for each case. Of the three placental specimens with evidence of involvement, the greatest dimension of involvement on MRI was 5, 12, and 24 mm, respectively. The median greatest dimension of involvement on MRI for the remainder of the cases that were negative on pathology was 15 mm (range: 11–20 mm).

In regard to neonatal outcomes, the overall neonatal composite occurred in 11 (28%) neonates, median APGARs at 1 min were 6 (median 6–8); no neonate had a pH < 7; and median birthweight was 2905 g (2721–3108 g). Ten (25%) infants were admitted to the NICU. Eight of these infants were born preterm and were sent to the NICU for either respiratory distress or prematurity (Table 3). The two infants born at term were sent to the NICU secondary to congenital anomalies (lumbosacral segmentation fusion defect and aortopulmonary window) and hypoglycemia monitoring due to maternal diabetes mellitus on insulin.

Twenty-one (52%) delivered preterm and 19 (48%) delivered at term. Of the cases that delivered preterm, 9 (43%) were delivered out of concern for vaginal bleeding. Of those 9 patients with vaginal bleeding leading to their preterm delivery, 7 (88%) experienced PPH at the time of delivery. Three of these seven patients (43%) with PPH required a transfusion. Other than vaginal bleeding, additional reasons for unscheduled preterm delivery included pre-labor rupture of membranes (*n* = 1), intrauterine fetal demise (*n* = 1), oligohydramnios (*n* = 1), vasa previa (*n* = 1), and preeclampsia with severe features (*n* = 1). Seven of 21 (33%) preterm deliveries were scheduled for PAS alone. All patients delivered preterm had a history of prior CD. Patients delivered preterm were more likely to have a placenta previa (76% vs. 42%, *p* = 0.0115) and were more likely to stay for more than 3 days (antepartum and postpartum stay included) in the hospital (24% vs. 0%, *p* = 0.049) (Tables 1 and 2). As could be predicted due to prematurity, neonatal length of stay was longer (6 [4–14] vs. 4 [4–4] days, *p* = 0.003) and the rate of the neonatal composite outcome was higher in patients delivered preterm (43% vs. 11%, *p* = 0.034). Neonatal birthweight, rate of NICU admission, and ventilatory support were not different (Table 3).

## 4 | DISCUSSION

Patients with prenatally suspected focal PAS as diagnosed on MRI had favorable delivery and neonatal outcomes, likely secondary to low rates of pathologically confirmed PAS and thus low rates of hysterectomy. Delayed delivery to the early term period was not associated with poorer maternal or neonatal outcomes.

Limitations of this study include its retrospective design and relatively small sample size. Further prospective studies with larger sample sizes are needed to make further recommendations for delivery timing in cases of focal PAS. Additionally, we did observe limited confirmation of focal PAS concern based on MRI to clinical FIGO (15%) and histopathology (23%). Pathologic examination is limited in its ability to assess for focal PAS. Sampling is performed at 5 cm increments, so despite best attempts to match the area of concern on imaging, there remains considerable potential to miss smaller pathologies. However, as all of these patients demonstrated concern for PAS on ultrasound, arguably the adjunct use of MRI was beneficial as it did help triage management that otherwise dictated even earlier delivery. As our understanding of this cohort expands, we will continue to search for imaging characteristics that may further differentiate clinical and histopathologic association. Finally, the improved delivery outcomes in cases of focal accreta delivered at term may be secondary to delivery urgency (scheduled vs. urgent/emergent), providing a source of selection bias.

Patients with prenatally suspected focal PAS generally have improved delivery outcomes. Preparation for possible hysterectomy and massive hemorrhage should still be taken in these cases, given an 8% rate of hysterectomy, 35% rate of transfusion, and 15% rate of positive pathology. Delivery at 37 weeks can safely be considered in cases of focal PAS at centers with access to serial sonographic imaging, adjunctive use of MRI, a uniform delivery protocol for patients with PAS, and a multidisciplinary team for management of such patients.

## Figures and Tables

**TABLE 1:** Delivery characteristics in cases of focal placenta accreta.

	All deliveries *N* = 40	<37 weeks *N* = 21	≥37 weeks *N* = 19	*p* value*
Placenta location				0.0115
Previa	24 (60)	16 (76)	8 (42)	
Low-lying	4 (10)	3 (14)	1 (5)	
Normal	12 (30)	2 (10)	10 (53)	
Uterine incision				0.629
Transverse	12 (30)	7 (33)	5 (26)	
Vertical	28 (70)	14 (67)	14 (74)	
Gynecologic oncology involvement				0.574
Readily available/planned for OR	33 (85)	3 (14.3)	4 (21.1)	
Present intra-op	2 (5)	0 (0)	2 (11)	0.127
Pathology				0.492
Negative	33 (85)	18 (90)	15 (79)	
BPMF	3 (8)	1 (5)	2 (11)	
Grade 2	1 (3)	1 (5)	0 (0)	
Grade 3A	1 (3)	0 (0)	1 (5)	
Grade 3E	1 (3)	0 (0)	1 (5)	

*Note*: Data reported as *N* (%). Statistical analysis included the chi-square and Fisher’s exact tests for categorical variables, Student’s *t*-tests for normally distributed continuous variables, and Kruskal–Wallis test for non-normally distributed continuous variables.

Abbreviations: BPMF, basal plate myometrial fibers without intervening decidua; OR, operating room.

* *p* value is a comparison of delivery before and at or after 37 weeks.

**TABLE 2:** Maternal outcomes in cases of focal placenta accreta.

	All deliveries *N* = 40	<37 weeks *N* = 21	≥37 weeks *N* = 19	*p* value*
Hysterectomy	3 (8)	1 (5)	2 (11)	0.596
Placental bed oversewn	6 (15)	4 (19)	2 (11)	0.664
EBL (mL)	1500 (1188–1812)	1500 (1250–2000)	1500 (1125–1625)	0.923
PPH	30 (75)	16 (76)	14 (74)	>0.999
Products transfused^a^				
PRBCs	12 (86)	7 (88)	5 (83)	>0.999
WB	3 (21)	2 (25)	1 (17)	>0.999
Platelets	2 (14)	1 (12)	1 (17)	>0.999
Plasma	4 (29)	2 (25)	2 (33)	>0.999
Postpartum LOS > 3 days	5 (12)	5 (24)	0 (0)	0.049

*Note*: Data reported as *N* (%), median (interquartile range), and median (range) for gestational age. Statistical analysis included the chi-square and Fisher’s exact tests for categorical variables, Student’s *t*-tests for normally distributed continuous variables, and Kruskal–Wallis test for non-normally distributed continuous variables.

Abbreviations: EBL, estimated blood loss; LOS, length of stay (days); PPH, postpartum hemorrhage (defined as >1000 cc at any delivery); PRBCs, packed red blood cells; WB, whole blood.

a Denominator for products transfused is total number transfused in each category.

* *p* value is a comparison of delivery before and at or after 37 weeks.

**TABLE 3:** Neonatal outcomes in cases of focal placenta accreta.

	All deliveries (*N* = 40)	<37 weeks (*N* = 21)	≥37 weeks (*N* = 19)	*p* value*
Birthweight (g)	2905 (2721–3108)	2840 (2580–2980)	2939 (2814–3188)	0.064
NICU	10 (25)	8 (38)	2 (11)	0.069
CPAP/Vent	4 (10)	4 (19)	0 (0)	0.108
Neonatal LOS (days)	4 (4–9)	6 (4–14)	4 (4–4)	0.003
Neonatal composite outcome^a^	11 (28)	9 (43)	2 (11)	0.034

*Note*: Data reported as *N* (%), median (interquartile range), and median (range) for gestational age. Statistical analysis included the chi-square and Fisher’s exact tests for categorical variables, Student’s *t*-tests for normally distributed continuous variables, and Kruskal–Wallis test for non-normally distributed continuous variables.

Abbreviations: CPAP/Vent, continuous positive airway pressure/ventilation; HIE, hypoxic ischemic encephalopathy; LOS, length of stay (days); NICU, neonatal intensive care unit.

* *p* value is a comparison of delivery before and at or after 37 weeks.

a Neonatal composite outcome includes cord pH < 7, cord base excess < −12, APGARs at 5 min ≤3, CPAP/ventilator support, HIE, birthweight <3rd percentile, NICU admission, and hypoglycemia.

## Data Availability

The data that support the findings of this study are available on request from the corresponding author. The data are not publicly available due to privacy or ethical restrictions.
